# The role of interleukin 10 in the development of the schizophrenia deficit syndrome in the light of the disconnection hypothesis

**DOI:** 10.1192/j.eurpsy.2023.1328

**Published:** 2023-07-19

**Authors:** P. Podwalski, E. Tyburski, A. Michalczyk, J. Samochowiec

**Affiliations:** 1 Department of Psychiatry; 2 Department of Health Psychology, Pomeranian Medical University, Szczecin, Poland

## Abstract

**Introduction:**

One of the subtypes of schizophrenia is the deficit syndrome (DS). Because of different risk factors, course, response to treatment weak prognosis, researches on this group of patients are important. Etiology of schizophrenia is often hypothesized as the inflammation process. Due to the imbalance of certain cytokines (interleukin-10 (IL-10) -antyinflamation cytokine, among others) changes in the function and structure of central nervous system may occur. That process could stand behind the outbreak of psychotic and deficit symptoms of the illness. Subinflammation can have an impact on the white matter structure. Disturbances in this area can cause impairment of cortical communication and hence, produce psychopathology. One of the structures that seem to have the basis of the deficit syndrome isinferior longitudinal fasciculus (ILF). ILF is a bundle of association fibers with interconnects temporal cortex with ocapital cortex.

**Objectives:**

The aim of our study was to investigate a relationship between the integrity of ILF and interleukin – 10.

**Methods:**

39 schizophrenia subjects divided into two groups DS (16) and non-deficyt sydrome (NDS) (23) and 18 healthy controls (HC) participated in the study. A DTI analysis was performed on all study participants. The psychopathology of schizofrenia was assessed using the Positive and Negative Syndrome Scale (PANSS). The ILF analysis was then conducted using fractional anisotropy (FA) and mean diffusivity (MD) parameters. Blood samples were obtained to analyze serum level of IL-10 level.

**Results:**

The differences in FA value in left ILF between DS and HC group were confirmed. The difference in values of IL-10 between groups were not confirmed. A negative correlation was found between FA values in left ILF and IL-10 (p = 0.033) among DS group.

**Conclusions:**

The imparment of the structure of ILF may be involved in ethiopatogenesis of DS. Moreover, changes in IL-10 levels may be related to the microstructure of ILF bundle.

**Disclosure of Interest:**

None Declared

